# Introducing a New Research Design: The Multidimensional Interpretative Textual (MIT) Design. Epistemological and Methodological Foundations

**DOI:** 10.1111/nin.70163

**Published:** 2026-08-30

**Authors:** Mariachiara Figura, Paola Arcadi, Ercole Vellone

**Affiliations:** ^1^ Department of Maternal and Child Health Promotion Internal Medicine & Specialties of Excellence ‘G. D'Alessandro’ (PROMISE) University of Palermo Palermo Italy; ^2^ Bachelor School of Nursing, ASST Melegnano e della Martesana University of Milan Milano Italy; ^3^ Department of Biomedicine and Prevention University of Rome ‘Tor Vergata’ Rome Italy

## Abstract

Contemporary nursing research increasingly generates large and complex textual corpora from interviews, open‐ended surveys, clinical documentation, policy discourse and professional narratives. Although these materials contain rich representations of professional reasoning, organisational dynamics and patient experiences, their analysis remains largely confined to thematic qualitative approaches or variable‐based quantitative models, which may struggle to examine how meanings are organised across extensive corpora. This article introduces the Multidimensional Interpretative Textual (MIT) design as an epistemological and methodological framework for corpus‐based discourse analysis in nursing research, rather than a specific analytical technique or software procedure. The MIT design conceptualises discourse as a structured empirical object and examines textual data at the corpus level. By integrating multidimensional statistical formalisation with interpretive reasoning, it renders patterns in the organisation of meanings empirically observable before theoretical significance is attributed to them. The article outlines the design's epistemological foundations, analytical object and research logic and proposes principles of methodological rigour, including analytical–interpretive separation, corpus structural adequacy, procedural traceability, interpretive arguability, structural–interpretive proportionality and theoretical interpretive transferability. The MIT design thus provides nursing science with a coherent research architecture for examining and comparing recurring patterns of meaning, professional reasoning and organisational logics across empirical contexts.

## 1 Background

Traditionally, textual data in healthcare research have been examined through qualitative methodologies grounded in interpretive traditions such as phenomenology, grounded theory and thematic analysis. These approaches emphasise close engagement with participants' accounts and the interpretive reconstruction of meaning within individual narratives or relatively small collections of texts. Through such methodologies, nursing scholarship has generated important insights into patients' lived experiences, professional identity formation and the relational dimensions of care.

The increasing availability of textual data has substantially expanded the analytical possibilities of healthcare research. Contemporary healthcare systems generate extensive collections of interviews, open‐ended survey responses, clinical documentation, policy texts, social narratives and professional discourses. As the scale and complexity of these materials increase, researchers face analytical questions that cannot be addressed solely through the interpretation of individual narratives. Large collections of textual data make it possible to examine how meanings recur, associate and vary across a corpus, creating the need for analytical approaches capable of investigating these broader patterns.

Within this context, statistical approaches to textual analysis have gained increasing relevance. Often referred to as lexicometry or statistical text analysis (Bolasco 2012; Lebart et al. 1998), these approaches conceptualise textual data as structured datasets, hereafter referred to as *textual corpora*. Rather than treating texts as isolated narratives, they examine the distribution and co‐occurrence of lexical units across a corpus to identify recurring patterns of association among meanings. The underlying assumption is that meaning is not only expressed through individual statements but also through the patterns of relationships that linguistic elements establish throughout the corpus. The objective is, therefore, not simply to count words, but to investigate how ideas, concepts and representations become organised through patterns of proximity, opposition and association among lexical elements. This relational perspective enables researchers to move beyond the interpretation of isolated texts and to explore broader semantic structures emerging across the corpus as a whole (Greenacre 2017; Reinert 1983, 2001).

A major development within this tradition has been the emergence of Exploratory Multidimensional Data Analysis (EMDA), which conceptualises discourse as a relational system rather than as a collection of independent texts (Fraire 2009). Through multidimensional statistical procedures, EMDA examines how lexical and contextual elements are positioned in relation to one another within a corpus, enabling the identification of broader patterns in the organisation of discourse. Meanings are therefore examined through patterns of proximity, opposition and association among lexical and contextual elements, making the discourse observable as an organised relational structure distributed across the corpus.

Empirical applications have demonstrated the analytical potential of these approaches across diverse healthcare contexts. Statistical textual analyses have been used to examine patient narratives, professional discourses, clinical documentation and free‐text survey responses, supporting the identification of shared representations and contextual variations within healthcare systems. For example, these approaches have been used to explore how nurses collectively represent concepts such as sustainability, professional autonomy or caregiving responsibilities across different healthcare settings, revealing recurrent semantic configurations and differences in the organisation of meanings across contexts (Bartoli et al. 2026; Figura et al. 2026; Khanbhai et al. 2021; MacLeod et al. 2024; Martínez‐Angulo et al. 2023; Rago et al. 2026).

## 2 Methodological Gap

Despite their growing use, the methodological status of statistical textual analysis within nursing research remains insufficiently clarified. While the analytical procedures themselves are well established, there is considerably less agreement regarding the nature of the analytical object they investigate, the type of knowledge they produce and the principles through which their findings should be interpreted and evaluated.

This situation reveals a methodological paradox. While the volume and complexity of available textual data have increased substantially, the epistemological frameworks guiding how such data should be analytically conceptualised and interpreted remain comparatively underdeveloped. In many studies, statistical techniques for textual analysis are applied primarily as technical tools for managing large textual datasets rather than as components of an explicitly articulated research design (Duchastel and Laberge 2019; Grimmer and Stewart 2013; Romano et al. 2020). As a result, statistical textual analyses are frequently interpreted through methodological frameworks developed for qualitative or mixed‐methods research, or, alternatively, treated as technical procedures for managing large textual datasets. Consequently, important methodological questions remain unresolved. What constitutes the analytical object of these studies? What type of knowledge do they produce? What is the epistemic status of statistically derived structures? And how should interpretation be conducted when discourse itself becomes the primary empirical object of investigation? These questions point to a broader methodological gap that has yet to be explicitly addressed within nursing research.

This ambiguity becomes particularly problematic when discourse is conceptualised not merely as a source of themes, categories or narratives, but as the primary empirical object of analysis (Anderson and Holloway 2020). In such cases, analytical outputs cannot be treated as self‐evident findings. Rather, they require explicit methodological principles governing interpretation, evaluation and theoretical integration. Without such principles, uncertainty emerges regarding what statistically derived structures represent, how rigour should be evaluated and what interpretive responsibilities belong to the researcher (Davidson et al. 2021).

Three interrelated methodological challenges follow from this situation. First, the epistemic status of statistically derived structures remains unstable, with analytical outputs sometimes treated as direct representations of discourse rather than analytical constructions that require careful interpretation (Baden et al. 2022; Hannigan et al. 2019; Isoaho et al. 2021). Second, criteria for evaluating rigour are frequently borrowed from qualitatively coded research and remain poorly aligned with the specific requirements of corpus‐based discourse analysis, including corpus construction, statistical formalisation and the assessment of structural stability (Montalescot et al. 2024; Traynor 2006). Third, the interpretive role of the researcher often remains insufficiently specified, making it difficult to understand how analytical structures are translated into meaningful nursing knowledge (Aranda et al. 2021; Denecke and Reichenpfader 2023; Duchastel and Laberge 2019).

These limitations extend beyond methodological concerns. When statistically formalised analyses remain insufficiently grounded within a clear epistemological framework, their contribution to nursing theory, professional reasoning and practice‐oriented knowledge becomes fragile. Analytical outputs risk remaining isolated technical artefacts rather than contributing to cumulative disciplinary knowledge capable of informing care, education and organisational decision‐making (Alejandro and Zhao 2024; Jacobs and Tschötschel 2019; MacLeod et al. 2024). For this reason, corpus‐based discourse research increasingly requires research designs capable of explicitly articulating the epistemological logic connecting statistical formalisation, interpretation, theory development and knowledge production. Such designs must clarify the analytical object under investigation, the status of statistically derived structures, the role of the researcher in interpretation, the contribution of theory to knowledge development and the criteria through which rigour can be evaluated.

To address this gap, this paper introduces the Multidimensional Interpretative Textual (MIT) design, a research design that conceptualises discourse as a primary empirical object and situates statistical formalisation within an explicitly interpretive methodological framework.

## 3 Definition of the MIT

The MIT design is a research design for corpus‐based discourse research in which discourse constitutes the primary empirical object of investigation. Starting from a corpus of textual data, the MIT design investigates how meanings become organised across the corpus as a whole. It is not defined by a specific analytical technique, software package or statistical procedure. Rather, it provides an epistemological and methodological framework that specifies how discourse is constituted as an analytical object, how it is formally organised through statistical procedures and how the resulting configurations are interpreted (Aranda et al. 2021; Duchastel and Laberge 2019; Jacobs and Tschötschel 2019).

Within the MIT design, discourse constitutes the analytical object, whereas the corpus provides the empirical space through which that object becomes observable. The corpus is not treated as a collection of independent texts, narratives or statements, but as an organised system of meanings distributed across discourse (Fraire 2009; Jacobs and Tschötschel 2019). Statistical formalisation makes corpus‐level configurations empirically observable before theoretical meaning is attributed to them. This formalisation is primarily achieved through procedures derived from statistical text analysis and EMDA traditions (Bolasco 2012; Fraire 2009), which organise textual productions into an analytical space defined by patterns of proximity, distance and opposition among lexical and contextual elements.

**Figure 1:**
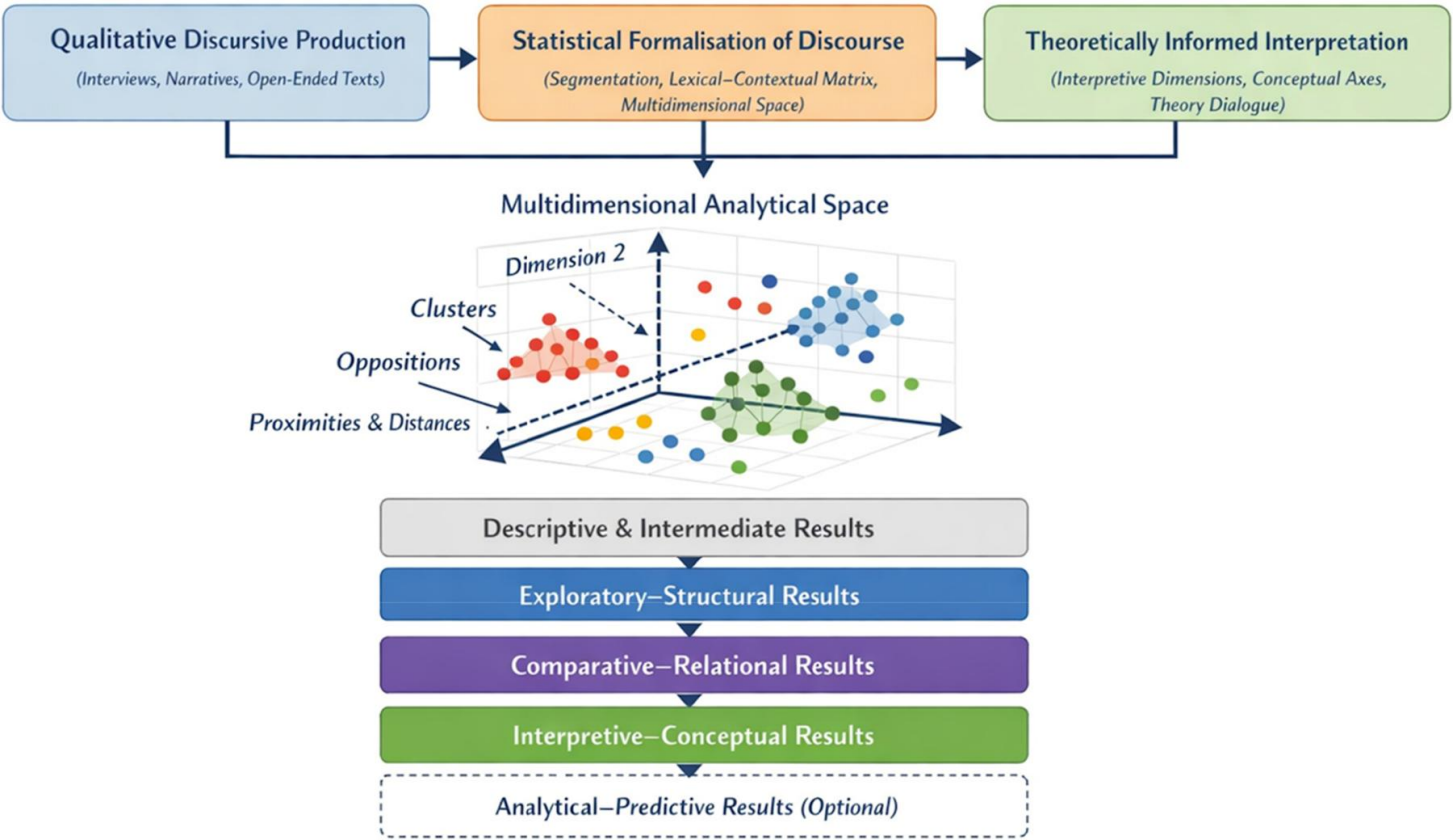
Epistemological logic of the MIT design. Conceptual overview illustrating the analytical sequencing underlying MIT, from qualitative discursive production to statistical formalisation and theoretically informed interpretation. The multidimensional analytical space represents how oppositions, clusters, proximities, and relational configurations delimit interpretive possibilities. Successive analytical layers illustrate the progression from descriptive and exploratory outputs toward relational and interpretive conceptual results.

Statistical formalisation does not assign interpretive meaning. Rather, it organises textual data into observable configurations that define the analytical space within which interpretation subsequently takes place. Statistical indicators function as empirical constraints on interpretation by showing how meanings are distributed, associated, differentiated and opposed across the corpus. Analytical procedures such as correspondence analysis, hierarchical clustering, multidimensional modelling and other statistical text analysis techniques are tools used within this process, but they do not define the MIT design itself. What characterises the design is the analytical sequence through which discourse is first formalised as an empirical structure and only subsequently interpreted (Figure 1).

The MIT design occupies a distinct position within the methodological landscape of nursing research. Qualitative approaches are particularly effective for exploring lived experiences, contextual meanings and narrative constructions. In many qualitative traditions, analysis proceeds from individual textual units toward progressively more abstract interpretive categories: researchers examine excerpts, narratives or statements, develop codes and subsequently construct themes through iterative engagement with the data. Within the MIT design, the analytical sequence is different. Rather than beginning with individual textual units and building upward toward thematic interpretations, MIT first formalises discourse at the level of the corpus as a whole, making its overall structure empirically observable. Interpretation then focuses on these corpus‐level configurations, examining how meanings are organised, associated, differentiated and opposed across discourse.

The distinction from quantitative research is equally important. Quantitative approaches investigate measurable relationships among predefined variables and use statistical procedures to estimate, compare or test those relationships. Although the MIT design also employs statistical procedures, statistics serve a different epistemological function. They are not used to measure attributes, quantify constructs, test hypotheses or establish relationships among variables. Instead, they formalise discourse by making corpus‐level configurations observable before interpretation. For this reason, the presence of statistical analysis does not make the MIT design a quantitative design.

The MIT design is also distinct from mixed‐methods research. Mixed‐methods designs combine distinct qualitative and quantitative forms of evidence within a single study, often for triangulation, complementarity or expansion. In contrast, the MIT design does not integrate separate qualitative and quantitative strands. Statistical formalisation and interpretation are sequential phases of a single analytical process directed toward the same object of inquiry: the organisation of meanings across discourse. Its distinctive contribution lies not simply in identifying meanings themselves, but in examining the discursive configurations through which meanings become organised and related across a corpus. What makes the MIT a research design rather than an analytical technique is that it does not merely provide procedures for analysing textual data. Rather, it defines the analytical object of inquiry, specifies the sequence linking statistical formalisation and interpretation, clarifies the role of theory and the researcher in knowledge production, and establishes principles through which rigour and interpretive claims can be evaluated. In this sense, the MIT offers a coherent research architecture linking epistemology, methodology, analysis and interpretation, thereby fulfilling the defining functions of a research design (Table 1).

**Table 1:** Epistemological and methodological distinctions between MIT and established research designs.

Dimension	Qualitative research	Quantitative research	Mixed‐methods research	MIT design
Primary analytical object	Lived experiences, narratives, meanings or social processes as expressed in textual units	Variables, measurable attributes and their relationships	Integration of qualitative and quantitative evidence	Organisation of meanings across discourse at the corpus level
Unit of analysis	Individual narratives, excerpts, interviews, observations or cases	Variables, observations, measurements or cases	Multiple units depending on the qualitative and quantitative components	Discursive configurations, latent dimensions and corpus‐level relational structures
Constitution of the analytical object	Progressively constructed through iterative interpretive engagement with data	Defined a priori through variables, constructs and measurement procedures	Constructed through the integration of qualitative and quantitative components	First formalised through corpus‐level statistical procedures and subsequently interpreted
Role of statistical procedures	Generally absent or ancillary	Measurement, estimation, hypothesis testing and modelling relationships among variables	Used within the quantitative component of the design	Formalisation of discourse and identification of corpus‐level configurations
Role of interpretation	Central to the construction of themes, categories and meanings	Primarily explanatory following statistical analysis	Integrates findings from qualitative and quantitative strands	Explains the significance of configurations revealed through statistical formalisation
Relationship between analysis and interpretation	Analysis and interpretation are closely intertwined throughout the research process	Interpretation follows statistical analysis	Analysis and interpretation occur within separate strands before integration	Statistical formalisation precedes and constrains interpretation
Primary analytical outputs	Themes, categories, concepts, narratives and theories	Estimates, associations, models, effects and predictions	Integrated findings and meta‐inferences	Discursive configurations, latent dimensions, oppositions and relational structures
Type of knowledge produced	Contextual and interpretive understanding of experiences and meanings	Explanatory and predictive knowledge about measurable phenomena	Integrated understanding derived from multiple forms of evidence	Structural and relational knowledge concerning the organisation of meanings across discourse
Primary research question	What meanings, experiences or processes are expressed by participants?	What relationships exist among measurable variables?	How can different forms of evidence be integrated to understand a phenomenon?	How are meanings organised, associated, differentiated and structured across discourse?

This analytical logic becomes evident in empirical applications. In a study exploring nurses' representations of environmental sustainability, statistical formalisation revealed that sustainability was organised differently across intensive care settings. Although similar themes appeared throughout the corpus, their organisation and relative salience varied according to clinical context. The analytical contribution of the study did not consist in identifying sustainability‐related themes per se, but in revealing how those themes were organised differently across clinical contexts (Bartoli et al. 2026). Similarly, a study investigating nursing students' representations of the humanised hospital revealed broader configurations of meaning and latent dimensions structuring how students collectively organised their understanding of humanised care across the corpus. Rather than simply identifying values associated with humanisation, the analysis made it possible to examine how those meanings were organised and related within the broader discursive structure (Lo Monaco et al. 2026).

The primary contribution of the MIT design is therefore not the identification of themes themselves, but the formalisation and interpretation of the discursive configurations through which meanings become organised across a corpus. Consequently, the unit of interpretation is not the isolated theme, narrative or statement, but the configuration of relationships through which meanings become structured across discourse (Bolasco 2012; Greenacre 2017; Reinert 1983, 2001).

Statistical formalisation may reveal latent dimensions and other discursive configurations, representing patterns of organisation that become observable only when discourse is examined at the level of the corpus. Latent dimensions are frequently expressed through recurrent polarities that structure the semantic space of the corpus (Fraire 2009; Reinert 2001). Their analytical value lies not in identifying new thematic content, but in revealing the configurations through which meanings become organised across discourse.

Interpretation consists of examining these empirically observable configurations through theoretical and contextual lenses, translating them into meaningful disciplinary knowledge. Statistical procedures identify the configurations; interpretation explains their significance. In this sense, interpretation remains grounded in observable corpus structures rather than relying exclusively on close reading of individual texts (Duchastel and Laberge 2019; Ricœur 1976, 1981).

**Figure 2:**
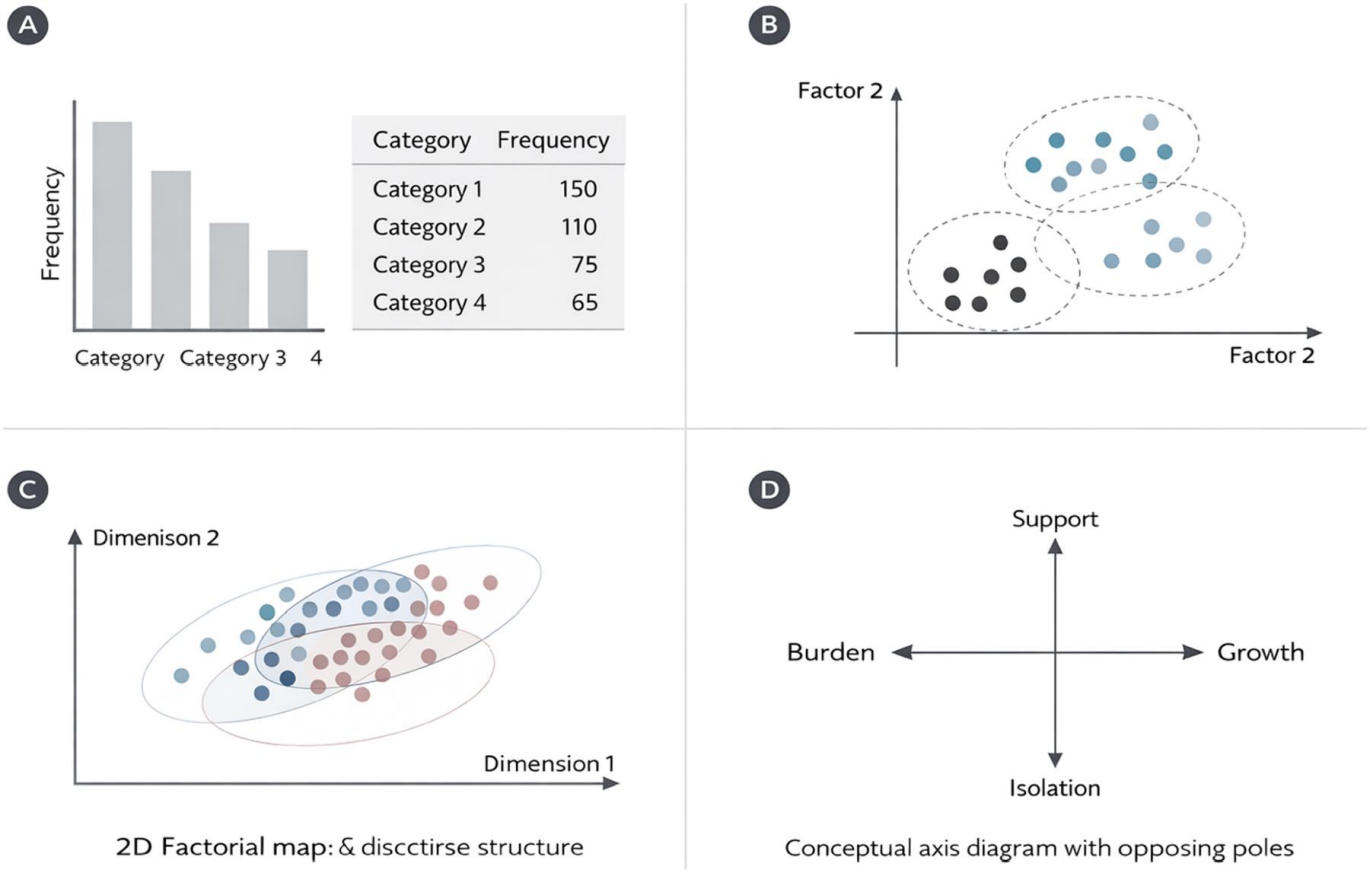
Stratified analytical outputs in MIT design. Illustration of different analytical outputs generated during multidimensional textual analysis. Panel (A) presents descriptive frequency‐based results. Panel (B) illustrates clustering within factorial space. Panel (C) represents discursive structuring within a two‐dimensional relational configuration. Panel (D) shows the conceptual abstraction of latent oppositional axes derived from structural interpretation.

Within the MIT design, statistical formalisation produces different types of analytical outputs corresponding to distinct levels of inquiry (Figure 2). Descriptive outputs support corpus exploration and transparency through indicators such as frequency distributions, lexical richness measures, co‐occurrence tables and corpus composition statistics. Structural outputs reveal how meanings are organised across the corpus through recurring groupings, associations, oppositions and latent dimensions. Comparative outputs examine how these structures vary across contexts, such as professional groups, clinical settings, geographical regions or temporal phases. Finally, interpretive outputs translate these structured configurations into theoretically meaningful explanations.

The MIT design, therefore, does not seek merely to describe textual content. Rather, it provides a framework for investigating how meanings become organised, differentiated and related across discourse, allowing researchers to move from textual data to the structural organisation of meaning and subsequently to theoretically informed interpretation.

## 4 Epistemological Status and Foundations of the MIT Design

The MIT design is grounded in an interpretive and textual epistemology that conceives meaning as constructed, situated and context‐dependent, and recognises text as the privileged site where meaning becomes observable through language (Gadamer 2006; Gee 2010; Ricœur 1981). Rather than analysing isolated excerpts, cases or predefined variables, the MIT design operates at the level of the corpus, formalising discourse into a relational structure prior to interpretation. The aim is not to reconstruct individual experiential narratives or to privilege participant voice as the final analytical endpoint, but to examine how meanings are collectively organised across discourse. Individual testimonies remain essential as empirical contributions to the corpus; however, analytical attention focuses on the relational configurations that emerge across participants rather than on singular narrative reconstruction.

Within this research design, meaning is neither treated as an objectively measurable property of discourse nor reduced to a purely subjective interpretive construction. Instead, meaning is understood as an emergent relational configuration shaped by the organisation of discourse across the corpus. The structures revealed through statistical analysis are neither objective facts nor arbitrary interpretations. Rather, they function as analytical representations of how discourse is organised, helping researchers make visible patterns that may not be immediately apparent through reading alone. Similar to a map that helps us navigate a territory without being the territory itself, these representations require theoretically informed and reflexively explicit interpretation (Duchastel and Laberge 2019; Ricœur 1981). Interpretation is conceived as a hermeneutic process that places structured textual configurations in dialogue with their historical and cultural contexts and with the researcher's horizon of understanding. Interpretive assumptions must therefore remain explicit and epistemically accountable.

Methodologically, the MIT design is anchored in EMDA and statistical text analysis, traditions that conceptualise text as a structured and relational empirical object rather than as narrative support (Fraire 2009). Statistical formalisation provides the conditions through which underlying patterns in the organisation of discourse become observable at the corpus level. Techniques such as correspondence analysis, clustering procedures and other multidimensional approaches help identify how meanings are organised and related across the corpus without reducing meaning to measurement (Benzécri 1973; Lebart et al. 1998; Reinert 2001).

The MIT design is not easily positioned within the conventional qualitative–quantitative distinction because its analytical object is neither predefined variables nor individual narratives, but the organisation of discourse across a corpus. Discourse retains its qualitative nature while being organised through statistical procedures that render latent structures visible. Statistical formalisation does not assign or validate meaning; it delimits the structural configuration of the discursive field, establishing the conditions under which interpretation can be argued and scrutinised (Figura et al. 2023; Grimmer and Stewart 2013).

Within the MIT design, induction and deduction assume complementary roles within a structured analytical sequence. Induction governs the emergence of discursive configurations through exploratory and unsupervised procedures, allowing relational patterns to arise without imposing a priori semantic categories (Benzécri 1973). Deduction informs corpus construction—for example through theoretically grounded metadata or contextual variables—and subsequently guides the interpretation of emergent structures without predetermining their content (Elo and Kyngäs 2008; Tizón‐Couto and Lorenz 2021).

Although the MIT design employs both computational and statistical tools, these terms refer to different aspects of the analytical process. Computational tools are used to process, organise and manage textual data, whereas statistical procedures are used to identify patterns and relationships within the corpus. Neither constitutes the epistemological foundation of the design. Rather, they provide the technical infrastructure through which discourse can be organised and examined. The epistemic status and theoretical significance of findings derive from the research design and from the researcher's interpretive responsibility. In this sense, algorithms help reveal patterns in the corpus, whereas meaning emerges through theoretically informed interpretation (Gillings et al. 2024; Jacobs and Tschötschel 2019).

Accordingly, the MIT design cannot be reduced to the application of computational tools or statistical procedures. These procedures operate within a broader epistemological architecture that defines how discourse becomes analytically observable and theoretically interpretable.

The MIT design, therefore, does not reduce interpretation to technique. Rather, it formalises the conditions that make disciplined, reflexive and theoretically grounded interpretation possible (Table 2).

**Table 2:** Epistemological status and foundations of the MIT.

Dimension	Position in MIT	Epistemological implication
Nature of meaning	Meaning is understood as an emergent relational configuration of the discourse across the corpus	Meaning becomes analytically observable through structural organisation before interpretation
Status of text	Text is analytically formalised as a corpus‐level relational empirical object through statistical organisation of discourse	Meaning emerges from relational configurations across the corpus rather than from isolated excerpts or narratives
Function of statistical formalisation	Statistical procedures organise discourse into observable relational structures without assigning interpretive meaning	Formalisation delimits the analytical object and establishes conditions for accountable interpretation
Role of interpretation	Theoretically informed hermeneutic interpretation grounded in formally delimited corpus structures	Interpretation translates structured discourse into disciplinary knowledge while remaining reflexively accountable
Role of theory	Theory guides corpus construction, analytical contrasts and interpretation without imposing semantic categories	Theory orients inquiry while remaining open to empirical transformation
Deductive reasoning	Theoretical interrogation of statistically formalised structures	Deduction operates after analytical delimitation of the object rather than predetermining meaning
Epistemological aim	Production of structural and relational knowledge about discourse	Knowledge emerges through interpretation constrained by empirically observable configurations

## 5 Research Questions in MIT

Within the MIT design, the formulation of research questions constitutes a central epistemic decision rather than a preliminary descriptive step. Because the MIT design examines discourse as an organised system of meanings rather than as a collection of individual accounts, research questions are explicitly oriented toward the analysis of discourse as a system. This epistemic positioning differentiates the MIT design from qualitative approaches that privilege close engagement with experiential accounts and from quantitative designs focused on measuring predefined variables or testing causal hypotheses (Creswell and Plano Clark 2018; Sandelowski 2010).

In the MIT design, research questions investigate how meanings are organised, connected, differentiated and related to empirical conditions such as clinical settings, organisational contexts, professional roles, temporal phases or socio‐cultural environments. The epistemic focus is therefore on the analysis of meaning as a pattern of relationships emerging across the corpus as a whole. In this sense, the MIT design can address the meanings attributed to a phenomenon, provided that these meanings are examined as discursive structures rather than primarily through individual narrative reconstruction. This orientation is consistent with structural and lexicometric traditions in textual statistics, which conceptualise meaning as arising from relational patterns within the corpus (Benzécri 1973; Lebart et al. 1998; Reinert 2001).

Accordingly, research questions in the MIT design are relational, multidimensional and corpus‐oriented, focusing on how meanings become structured within discourse and how these structures relate to empirical contexts. They may concern the internal organisation of a single discursive space or comparative variations across contexts, populations or temporal phases. Typical examples include inquiries into how a concept is organised within a professional field, how a phenomenon is collectively represented within a corpus, which oppositions or tensions structure discourse around a given issue, how discourses vary across groups or contexts, and how discursive patterns are shaped by their organisational, professional, or social environments (Table 3). For example, in nursing research, the MIT design may investigate how professional autonomy is structurally configured across nurses' discourses in different organisational settings, or how sustainability is collectively represented within intensive care nursing discourse through recurring relational oppositions and semantic configurations.

**Table 3:** Example of research question typologies in MIT.

Type of research question	Analytical focus	Example formulation
Structural organisation	Internal architecture of discourse	How is the concept of care discursively structured within nursing narratives?
Relational polarities	Latent oppositions and tensions	Which latent polarities organise discourse around professional responsibility?
Collective representation	Share discursive configurations	How is patient safety collectively represented within a clinical corpus?
Corpus structuring	Latent dimensions organising discourse	Which latent dimensions organise discourse around patient autonomy in ICU settings?
Comparative variation	Differences across contexts or groups	How does discourse on burnout differ between junior and senior professionals?
Temporal transformation	Evolution of discursive structures	How has the representation of telemedicine evolved across time?
Contextual embedding	The relation between discourse and environment	How do organisational settings shape discursive constructions of teamwork?
Conceptual emergence	Formation of theoretical dimensions	Which latent dimensions organise discourse on dignity in care?

The MIT design is therefore indicated when the epistemic objective concerns the structural organisation of meaning within discourse and its relation to empirical contexts, rather than the measurement of variables or the saturation of thematic categories.

## 6 Role of Theory and Research Hypotheses in the MIT Design

In the MIT design, theory does not operate as a set of predefined categories imposed on the corpus, nor as a framework that determines in advance which meanings should emerge. Rather, it provides a conceptual orientation that guides how discourse is approached, delimited, and interpreted throughout the research process. Theory defines the analytical horizon within which inquiry unfolds, indicating where and how analysis should be directed while remaining open to empirical transformation (Aranda et al. 2021; Gillings et al. 2024).

At the stage of formulating research questions, theory supports the delimitation of the phenomenon under investigation by identifying analytically relevant contextual dimensions, such as clinical settings, organisational contexts, professional roles or temporal frames. Its function is therefore to clarify the boundaries of the analytical field rather than to prescribe the semantic content of discourse.

Within the MIT design, theory may assume different roles depending on the purpose of the study. In exploratory applications, theory primarily provides a general conceptual orientation and becomes most explicit during interpretation. In theory‐informed applications, theory may also contribute to corpus construction, the selection of contextual variables, metadata design or comparative dimensions. However, regardless of when theory enters the research process, it does not predetermine the organisation of meanings that emerge through statistical formalisation of the corpus. Rather, theory guides the investigation of discourse while remaining open to the empirical structures that emerge from the data.

Within the MIT design, research hypotheses are admissible only when they concern relational expectations between discursive configurations and contextual or categorical variables. Hypotheses do not predict the semantic content of discourse; instead, they formulate expectations about how discursive structures may differ, align, cluster or polarise across contexts. For example, a researcher may hypothesise that nurses working in different organisational settings will organise a phenomenon in different ways, without predicting in advance which meanings will emerge. In this sense, hypotheses concern relations between configurations rather than thematic content, preserving openness to empirical emergence (Grimmer and Stewart 2013).

During analysis, discursive configurations emerge through statistical formalisation of the corpus. Theory and hypotheses do not generate these structures; rather, they guide the selection of contextual variables, contrasts and relational comparisons through which the configurations are examined. Statistical formalisation reveals how discourse is organised across the corpus, while theoretical orientation ensures coherence and transparency in the interpretation of emergent structures.

Theory assumes its most explicit role at the interpretive stage. Statistical outputs such as dimensions, clusters or differences observed across groups and contexts do not possess intrinsic theoretical meaning and therefore require conceptual translation. Interpretation involves a theoretically informed dialogue between structured empirical configurations and relevant conceptual frameworks. In this process, empirical findings may refine, extend, or critically interrogate existing theoretical perspectives (Aranda et al. 2021; Duchastel and Laberge 2019; Tavory and Timmermans 2014).

Theory generation in the MIT design does not consist of producing new thematic categories through inductive coding. Instead, it arises from the conceptual articulation of recurrent relational configurations identified at the structural level of discourse (Aranda et al. 2021; Duchastel and Laberge 2019; Ricœur 1976). When such configurations demonstrate coherence, stability and recurrence across contexts, they may support the development of mid‐range theoretical propositions concerning how phenomena become discursively organised (Merton 1968; Tavory and Timmermans 2014). Theoretical advancement, therefore, occurs through the abstraction of relational dynamics rather than through the aggregation of textual fragments.

Empirical exploration and theoretical reflection thus operate in a sequential yet dialogical relationship: structural modelling precedes and constrains interpretation, while interpretation translates structured configurations into theoretically meaningful knowledge without predetermining their content.

## 7 Role of the Researcher in the MIT Design

Within the MIT design, the researcher occupies a distinct epistemic position that differs both from that of a neutral observer and from that of a mere operator of automated procedures. Patterns in the organisation of discourse emerge through statistical and computational analyses; however, their transformation into scientifically meaningful knowledge depends on theoretically informed interpretation. The MIT design, therefore, does not seek to minimise interpretation, but to render it explicit, arguable and epistemically accountable (Aranda et al. 2021; Gadamer 2006).

The researcher's responsibility extends across the entire research process and assumes different forms at each stage. In the initial phase, the researcher defines the analytical perspective and identifies the contextual dimensions through which discourse will be examined. The selection of clinical, organisational, categorical or temporal variables represents an epistemological decision that shapes the type of knowledge the study aims to produce. These decisions delimit the discursive field under investigation without intervening in the semantic organisation of the corpus.

During the analytical phase, patterns and structures in discourse become observable through formalised statistical procedures applied to the corpus. Nevertheless, these procedures depend on methodological decisions that remain under the researcher's responsibility, including corpus construction, text preparation procedures, segmentation of textual units and analytical formalisation choices. The researcher, therefore, remains accountable for the analytical conditions through which discursive configurations become observable.

Interpretation becomes most explicit after structural configurations have been identified. Analytical outputs such as clusters of discourse, relational profiles or latent dimensions that summarise broader patterns of variation across the corpus do not possess intrinsic theoretical meaning and require conceptual translation. Interpretation involves placing these structured configurations in dialogue with relevant theoretical frameworks and with the contextual conditions from which the corpus was produced. Labels attributed to emergent structures function as conceptual mediations that render findings communicable within the disciplinary field.

Within the MIT design, computational mediation does not diminish the researcher's role but redefines it. The researcher remains responsible for ensuring coherence between statistical formalisation, theoretical interpretation and the scientific claims derived from discourse (Duchastel and Laberge 2019; Ricœur 1976, 1981). Accordingly, the MIT design cannot be reduced to the application of computational tools or statistical procedures. These procedures operate within a broader epistemological architecture that defines how discourse becomes analytically observable and theoretically interpretable.

## 8 Principles of Rigour in the MIT Design

Rigour in the MIT design cannot be directly borrowed from traditional qualitative or quantitative frameworks because the analytical object differs from both. In MIT, the analytical object is the organisation of meanings across a corpus, made observable through statistical formalisation. Rigour must therefore be defined in relation to how patterns in discourse emerge, how interpretation is constrained by these patterns and how analytical decisions remain transparent and inspectable. The following principles are proposed as criteria for evaluating rigour in MIT studies and are intended to guide both the conduct and appraisal of research adopting this design.

### 8.1 Analytical–Interpretive Separation

Analytical construction and interpretation must remain explicitly separated. Statistical procedures first identify patterns in how meanings are organised across the corpus, whereas interpretation takes place only after these patterns have been established. Analytical outputs delimit the interpretive space; they do not contain interpretation in themselves. This separation prevents the premature attribution of meaning during analysis. If interpretation begins before patterns have stabilised, analytical decisions may unconsciously adapt to confirm emerging expectations. Rigour, therefore, requires that patterns of organisation within discourse become observable before they are interpreted (Greckhamer and Cilesiz 2014; Hannigan et al. 2019).

### 8.2 Corpus Structural Adequacy

A corpus must be capable of sustaining structural analysis. Adequacy does not refer to size alone: a large corpus may still be analytically weak. What matters is whether the corpus exhibits sufficient lexical repetition, distributional balance and internal coherence to generate stable patterns of organisation. For example, a corpus in which key meanings appear only sporadically may not support robust structural interpretation. A corpus is considered adequate insofar as it enables patterns in discourse to emerge consistently at the level required by the research question. Weak or fragmented patterns support only cautious descriptive claims, whereas robust and recurrent patterns justify broader theoretical articulation. Interpretive scope must therefore remain proportionate to the observable strength of the corpus‐derived patterns (Bolasco 2012; Lebart et al. 1998).

### 8.3 Procedural Traceability

The analytical process must remain traceable from corpus construction to interpretive conclusion. Corpus preparation, inclusion criteria, text preparation procedures, segmentation of textual units and analytical formalisation choices constitute epistemic decisions that shape what the corpus can reveal. These decisions must be documented. Traceability prevents the analytical workflow from becoming a black box and allows readers to reconstruct how the analysis was conducted. Rigour requires that analytical pathways remain inspectable and arguable, enabling critical appraisal of the adopted analytical logic and supporting cumulative knowledge production even in the absence of interpretive uniformity (Grimmer and Stewart 2013; van Atteveldt et al. 2022).

### 8.4 Interpretive Arguability

Interpretations must be theoretically informed, explicitly grounded in observable patterns in discourse, and supported by quantitative indicators that delimit the interpretive field. The existence of alternative readings is not excluded; rather, interpretive choices must be justified, discussed and situated within relevant theoretical traditions. Interpretive plurality is permitted, but interpretive arbitrariness is not: all readings must remain constrained by observable patterns of relationships within the corpus. Within this framework, arguability replaces replicability as the primary criterion of interpretive rigour: interpretations are evaluated in terms of their coherence, explanatory power and theoretical accountability rather than their uniqueness (Isoaho et al. 2021). Participant validation, when feasible, may function as a dialogical extension of interpretation rather than as a criterion of structural validation. Because patterns of organisation emerge at the level of the corpus rather than at the level of individual participants, no single participant can confirm or invalidate the analytical configuration. Participant feedback may enrich interpretive dialogue, but it does not constitute a methodological requirement or a condition of validity within the MIT design. The MIT design, therefore, does not seek interpretive objectivity in a positivist sense, but interpretive accountability grounded in observable patterns of discourse and theoretically explicit reasoning.

### 8.5 Structural–Interpretive Proportionality

Interpretive claims must remain proportional to the scale and strength of the analytical structure. Patterns identified in the corpus support conclusions only within the limits of what can be clearly observed in the data. When patterns are weak, unstable or poorly differentiated, conclusions must remain cautious and primarily descriptive. Broader theoretical articulation becomes legitimate only when patterns are robust, recurrent and clearly organised across the corpus. This principle regulates the scope of inference by aligning theoretical ambition with empirical visibility and preventing interpretive overreach (Greckhamer and Cilesiz 2014).

### 8.6 Theoretical Interpretive Transferability

What can be transferred across contexts is not the statistical structure itself but the interpretations developed from observable patterns in discourse. Rigour, therefore, requires that interpretations be articulated at a level of conceptual abstraction that allows their analytical translation into comparable empirical settings. Transferability is achieved through theoretical explicitation and structural description rather than through statistical generalisation (Duchastel and Laberge 2019; Lincoln and Guba 1986; Ricœur 1976).

Because the MIT design formalises discourse into a multidimensional relational space before interpretation, analytical outputs such as latent oppositions (recurring tensions between different ways of representing a phenomenon), relational proximities or discursive polarities function as structural constraints rather than transferable findings. These structures do not travel across contexts as empirical results. Rather, what may be transferable are the interpretive insights developed from them when comparable patterns of organisation emerge in other corpora. For example, studies exploring nurses' experiences in different institutional contexts may reveal similar latent oppositions, such as autonomy versus institutional constraint. Such recurrence does not imply identical thematic content but indicates analogous relational dynamics within discourse. In this sense, the MIT design supports the circulation of interpretive insights that remain anchored in discourse while retaining relevance beyond the original corpus (Table 4).

**Table 4:** Principles of rigour in the Multidimensional Interpretative Textual (MIT) design.

Principle	Core rule	Core question	Practical implication for researchers
1. Analytical–interpretive separation	Analysis must stabilise structures before interpretation begins	Am I interpreting after the structure exists, or shaping the structure to fit my expectations?	Complete statistical modelling first. Delay conceptual labelling until configurations are stable and observable. Avoid adjusting analytical parameters to confirm emerging interpretations.
2. Corpus structural adequacy	The corpus must sustain stable relational structures	Is the corpus strong enough to support the claims I want to make?	Evaluate lexical balance, repetition and internal coherence before interpretation. Treat weak structures cautiously and limit interpretive ambition when patterns are fragmented.
3. Procedural traceability	The analytical workflow must remain inspectable	Can another researcher reconstruct how this analysis was built?	Document corpus preparation, preprocessing, segmentation and analytical parameters. Treat every decision as an epistemic choice that must be explainable.
4. Interpretive arguability	Interpretations must be theoretically grounded and publicly arguable	Can readers see how I moved from structure to meaning?	Make theoretical positioning explicit. Show how interpretations derive from observable configurations. Allow competing readings, but anchor all claims to the same structural evidence.
5. Structural–interpretive proportionality	Interpretive scope must match structural strength	Am I claiming more than the structure allows?	Calibrate conclusions to the stability of the configuration. Weak structures justify description; robust structures justify broader theoretical articulation. Avoid overreach.
6. Theoretical interpretive transferability	Interpretations travel as models, not population claims	Can this interpretive model illuminate other contexts without claiming generalisation?	Articulate findings at a conceptual level that allows comparison. Describe structural boundaries clearly so others can judge relevance through analogy, not statistics.

## 9 What Type of Knowledge Does MIT Produce?

**Figure 3:**
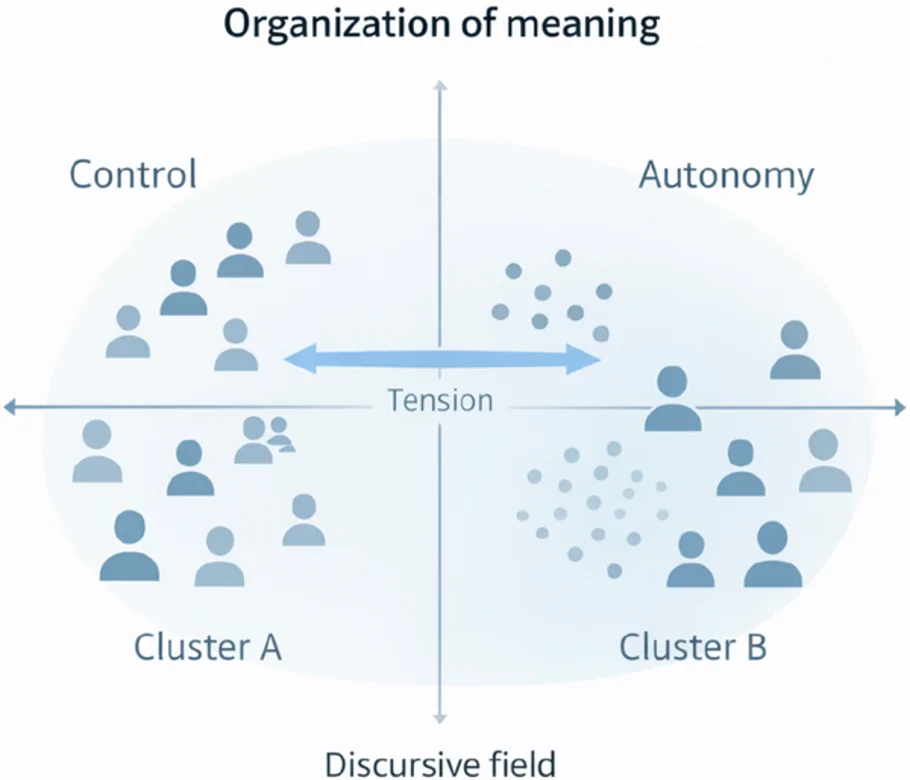
Organisation of meaning according to the MIT design. Conceptual representation of how meaning emerges within a relational discursive space through tensions between opposing poles. Clusters illustrate groups of discursive positions organised along latent dimensions, highlighting how interpretive meaning arises from relational configurations rather than isolated textual segments.

The MIT design produces structural and relational knowledge about discourse. Unlike qualitative approaches that identify themes or quantitative designs that measure predefined variables, MIT makes visible how meanings are organised across a corpus by rendering patterns of organisation empirically observable before interpretation attributes theoretical significance to them. The knowledge generated, therefore, concerns the patterned organisation of discourse rather than isolated statements or individual perspectives. These patterns may take the form of latent dimensions, discursive polarities or recurrent oppositions in meaning. Latent dimensions are underlying patterns of organisation that are not directly observable through the reading of individual texts but become visible when discourse is analysed at the level of the corpus. Such dimensions often emerge as polarities, that is, recurring tensions between different ways of representing, understanding or experiencing a phenomenon (Figure 3) (Benzécri 1973; Reinert 1983, 2001).

These configurations reveal tensions and structured positions through which phenomena are collectively articulated. The analytical focus lies in understanding how meanings become organised, differentiated, and stabilised within discourse (Duchastel and Laberge 2019; Ricœur 1976). For example, in nursing research, the MIT design may reveal how organisational discourse surrounding patient safety becomes structured around recurrent tensions between institutional standardisation and professional autonomy, or how sustainability discourse in intensive care settings polarises around competing meanings of responsibility, feasibility and resource management.

This orientation has implications for transferability. Findings are not transferred as empirical generalisations or thematic summaries, but as interpretations of recurring patterns in how meanings are organised. What becomes comparable across contexts is not specific content but the relational patterns organising discourse. Such patterns may reappear or transform in other settings, allowing theoretically informed comparison across studies (Duchastel and Laberge 2019; Lincoln and Guba 1986; Ricœur 1976).

The MIT design, therefore, supports cumulative knowledge production grounded in the comparison of patterns in discourse across studies and contexts. The knowledge produced is neither idiographic nor statistically generalisable; rather, it is structurally comparable because it concerns how meanings are organised rather than the specific content through which they are expressed. Its scientific value lies in making the organisation of meaning empirically observable and theoretically interpretable within nursing research (Figura et al. 2023; Sandelowski 2010).

## 10 Positioning and Innovative Relevance of the MIT Design in Nursing Research

The MIT design is positioned as an epistemological and methodological framework within the methodological landscape of nursing and health sciences. Its contribution does not consist in introducing new computational techniques or statistical procedures, but in providing a coherent research design capable of governing how statistical formalisation, interpretation and theoretical explanation interact within corpus‐based discourse research. The MIT design responds to a specific analytical need that has become increasingly visible in contemporary nursing research: understanding how meanings are organised across large textual corpora rather than solely within individual narratives, themes or variables.

Contemporary nursing and health research increasingly generates extensive collections of textual materials, including interviews, open‐ended survey responses, clinical documentation, policy documents and professional narratives. These materials often contain collective representations, professional reasoning, organisational tensions and shared understandings that extend beyond the level of individual accounts. While qualitative approaches are particularly effective for exploring experiences and meanings within specific contexts, and quantitative approaches are designed to investigate relationships among predefined variables, neither was originally developed to examine how meanings become organised across discourse at the level of an entire corpus. The MIT design addresses this analytical challenge by providing a framework for investigating the organisation of meanings across large collections of texts.

Within health research, textual data have traditionally been associated with qualitative inquiry, whereas statistical procedures have primarily been linked to measurement and inference (Figura et al. 2024; Jacobs and Tschötschel 2019). Although lexicometric and multidimensional textual traditions have long demonstrated the analytical value of statistical formalisation for examining discourse, these approaches have rarely been articulated as explicit research designs governing epistemological positioning, interpretive accountability and criteria of methodological rigour within nursing research (Benzécri 1973; Duchastel and Laberge 2019; Reinert 2001). As a result, multidimensional textual analyses are often applied as analytical tools embedded within existing methodological traditions rather than as components of a coherent research design.

The MIT design addresses this methodological gap by providing an explicit framework that clarifies how statistical formalisation, interpretation and theoretical explanation interact in corpus‐based discourse research. Its purpose is not to retrospectively relabel existing corpus‐based studies, nor to suggest that multidimensional textual analyses have not previously generated valuable knowledge. Rather, the MIT design seeks to provide an explicit epistemological and methodological framework capable of explaining how such analyses produce knowledge, how interpretation should be conducted and how rigour can be evaluated.

The distinctive contribution of the MIT design lies in its analytical sequence. Rather than progressively constructing the analytical object through excerpts, cases or narratives, MIT first formalises discourse at the corpus level and subsequently interprets the resulting configurations. This enables researchers to investigate not only what meanings are expressed but also how they are organised and related across discourse.

The conceptual development of the MIT design is informed by empirical studies employing multidimensional textual analyses in nursing and health sciences. Across domains such as critical care practice, transitional care, migrant health, sustainability and professional development, these studies have demonstrated the analytical value of examining discourse as an organised field of meanings in which professional, organisational and experiential perspectives intersect. Rather than simply cataloguing experiences or themes, this body of work has shown how recurring patterns in discourse can reveal broader structures through which phenomena are collectively understood and represented. The MIT design builds upon this accumulated empirical experience by providing a coherent framework capable of integrating statistical formalisation, interpretation and theoretical development within nursing research (Bolasco 2012; Figura et al. 2023; Fraire 2009).

## 11 Conclusions

This article has introduced the MIT design as an epistemological and methodological framework for the structural analysis of discourse in nursing research. By conceptualising discourse as a primary empirical object and positioning statistical formalisation within an explicitly interpretive framework, the MIT design provides a coherent approach for examining how meanings become organised across textual corpora.

Contemporary healthcare systems generate increasingly large and complex bodies of textual data, including interviews, clinical documentation, policy discourse, professional narratives and open‐ended inquiry materials produced across educational, organisational and clinical contexts. These materials often contain collective representations, professional reasoning, ethical tensions and organisational logics that extend beyond the analytical reach of thematic description or variable‐based modelling.

For nursing research, this means not only identifying what professionals, patients or organisations say about a phenomenon but also investigating how meanings become collectively structured, negotiated and stabilised across discourse. In this sense, the MIT design enables the investigation of recurring tensions underpinning healthcare systems, such as autonomy and institutional constraint, standardisation and professional judgement or responsibility and organisational feasibility.

By making patterns in the organisation of discourse empirically observable, the MIT design enables researchers to investigate how professional reasoning, organisational priorities and care practices are collectively articulated within healthcare systems. The knowledge produced through this approach concerns the organisation of meanings across discourse and supports cumulative inquiry through the comparison of recurring patterns across studies and contexts rather than through statistical generalisation.

The MIT design should therefore be understood not as a technical extension of textual statistics, but as a research design that establishes the epistemological and methodological conditions through which discourse can be structurally analysed and theoretically interpreted within nursing research.

The development of the MIT design contributes to expanding the methodological repertoire of nursing research by providing a framework capable of integrating statistical formalisation and interpretive reasoning within corpus‐based discourse analysis. Future research will be required to apply the MIT design across diverse empirical domains, refine reporting standards and further develop criteria of rigour appropriate for structurally formalised discourse analysis.

Rather than a closed methodological system, the MIT design should be viewed as an evolving research programme aimed at strengthening how nursing research investigates, interprets and theorises the organisation of meanings within discourse.

## Author Contributions

Conceptualization: M.F., P.A. and E.V. Methodology: M.F., P.A. and E.V. Writing—original draft: M.F. and P.A. Writing—review and editing: M.F., P.A. and E.V. Supervision: E.V. Project administration: M.F. All authors have read and approved the final manuscript.

## Funding

The authors have nothing to report.

## Ethics Statement

This manuscript presents a methodological and conceptual research design describing the Interpretive Multidimensional Textual (MIT) research design. As the article does not involve the collection or analysis of data from human participants, ethical approval was not required according to institutional and international research ethics guidelines.

## Conflicts of Interest

The authors declare no conflicts of interest.

## Artificial Intelligence (AI) Disclosure

During the manuscript preparation, the authors used OpenAI's ChatGPT to assist with language editing and readability. The AI tool was not used to generate data, perform analyses, produce results, or formulate scientific conclusions.

## Data Availability

Data sharing is not applicable to this article as no datasets were generated or analysed during the development of this methodological and conceptual work.
